# Quizartinib (AC220) reverses ABCG2-mediated multidrug resistance: *In vitro* and *in vivo* studies

**DOI:** 10.18632/oncotarget.21078

**Published:** 2017-09-16

**Authors:** Jun Li, Priyank Kumar, Nagaraju Anreddy, Yun-Kai Zhang, Yi-Jun Wang, Yanglu Chen, Tanaji T. Talele, Kanav Gupta, Louis D. Trombetta, Zhe-Sheng Chen

**Affiliations:** ^1^ Department of Pharmaceutical Sciences, College of Pharmacy and Health Sciences, St. John’s University, Queens, New York, 11439, USA; ^2^ Department of Otolaryngology-Head and Neck Surgery, Zhongnan Hospital of Wuhan University, Wuhan, 430071, China; ^3^ Department of Chemistry, Princeton University, Princeton, NJ, 08544, USA; ^4^ Jericho High School, Jericho, NY, 11753, USA

**Keywords:** quizartinib, ABCG2, ABCC1, MDR, reversal

## Abstract

Previous reports have shown that some tyrosine kinase inhibitors (TKIs) could inhibit the ATP-binding cassette (ABC) transporters involved in multidrug resistance (MDR). Quizartinib (AC220), a potent class III receptor tyrosine kinase inhibitor (TKI), was synthesized to selectively inhibit FMS-like tyrosine kinase-3 (FLT3), a target in the treatment of acute myeloid leukemia (AML). Quizartinib is currently under clinical trials for *FLT3* ITD and wild-type AML and is tested in combination with chemotherapy. While non-toxic to cell lines, quizartinib at 3 μM showed significant reversal effect on wild-type and mutant ABCG2 (R482T)-mediated MDR, and only a moderate reversal effect on mutant ABCG2 (R482G)-mediated MDR. Results also showed that quizartinib reversed MDR not by reducing the expression of ABCG2 protein, but by antagonizing the drug efflux function and increasing the intracellular accumulation of substrate anticancer drugs in ABCG2-overexpressing cells. Importantly, quizartinib at 30 mg/kg strongly enhanced the effect of topotecan (3 mg/kg) in ABCG2-overexpressing (H460/MX20) xenografts in athymic nude mice. These results demonstrated that quizartinib potentiates the antineoplastic activity of wild-type and R482T mutant ABCG2 substrates. These findings may be useful in clinical practice for cancer combination therapy with quizartinib.

## INTRODUCTION

During the course of chemotherapy treatment, cancer cells develop resistance to anticancer drugs by either intrinsic or acquired mechanisms which lead to the development of multidrug resistance (MDR) [1, 2]. MDR is a phenomenon in which cancer cells exhibit simultaneous resistance to anticancer drugs that have different structures and mechanisms of action [1, 3]. MDR results in decreased efficacy of anticancer drugs [2, 4].

The proposed mechanisms for MDR include alteration in the permeability of lipid bilayer membrane, inhibition of apoptosis, increased DNA repair of cancer cells, decreased inactivation/detoxification of drugs, changes in the number of cell surface receptors, overexpression of transporters which efflux drugs out of the cells, or a combination of one or more of these above mentioned factors [5, 6]. Cancer cells typically have the intrinsic property to develop drug resistance, but the use of chemotherapy allows cancer cells to develop resistance towards a broad spectrum of drugs [7]. Usually, this acquired resistance to a broad spectrum of anticancer drugs is due to the overexpression of energy dependent efflux proteins, the ATP-binding cassette (ABC) transporters that pump out drugs from cells against a concentration gradient [3, 8–10].

The ABC transporter family is one of the largest transmembrane protein superfamilies [11]. It is diverse and ubiquitously present in both prokaryotes and eukaryotes. Currently, 49 human ABC transporters have been identified, of which 48 members are functional while ABCC13/MRP10 is considered nonfunctional [9, 10, 12, 13]. The ABC transporter subfamily B member 1 [ABCB1, also known as Multi-Drug Resistance 1 (MDR1)/ P-glycoprotein (P-gp)], ABC transporter subfamily C member 1 [ABCC1, also known as Multidrug Resistance Protein 1 (MRP1)] and ABC transporter subfamily G member 2 [ABCG2, also known as Breast Cancer Resistance Protein (BCRP)/ MitoXantrone Resistance protein (MXR)/ ATP-Binding Cassette of Placenta (ABCP)] appear to promote MDR in cancer cells [1, 2, 7, 12, 14].

Although challenging, current research interests are primarily focused on the development of novel compounds that are selective, non-toxic, and effective against MDR malignancies. Recently, it has been shown that tyrosine kinase inhibitors (TKIs), at clinically achievable concentrations, can inhibit the ATPase activity of ABC transporters, inhibit active drug efflux, and overcome drug resistance in cells that develop the MDR phenotype as a result of overexpressing ABC transporters [15–17]. Indeed, MDR mediated by ABCB1 and ABCG2 can be reversed by 1) EGFR inhibitors gefitinib (ZD-1839), erlotinib (OSI-774) and AG1478; 2) EGFR and HER-2 inhibitor lapatinib (GW-572016); pan-HER inhibitor canertinib (CI-1033); 3) BCR-ABL inhibitor imatinib (STI-571) and 4) certain multi-kinase inhibitor such as sunitinib (SU-11248) [16, 18–21]. We have reported that the TKIs erlotinib, lapatinib, imatinib, nilotinib and ponatinib significantly potentiated the cytotoxicity of paclitaxel, vincristine, and other chemotherapeutic drugs by blocking ABCC10-mediated MDR [22–24]. These TKIs are used clinically in the treatment of various cancers and they could be used as MDR reversal agents in combination with conventional antineoplastic drugs.

Quizartinib is a potent and selective second generation, small molecule class III receptor tyrosine kinase inhibitor with better pharmaceutical properties and superior pharmacokinetic profile. Studies have demonstrated that quizartinib has high efficacy and tolerability in tumor xenograft models that express *FLT3*-ITD mutant kinase [25–27]. Quizartinib inhibits cellular FLT3 autophosphorylation and cell viability in MV4-11 cells overexpressing activated FLT3 [27]. Quizartinib inhibits cellular signaling in wild type as well as ITD-overexpressing cells, and it induces apoptosis in cells that have constitutively activated FLT3 [27]. It is currently under clinical trial for acute myeloid leukemia by Ambit biosciences (http://www.ambitbio.com/clinical_trials). Furthermore, quizartinib does not add toxicity when combined at monotherapy dose with other chemotherapeutic drugs [28]. In this study we examined the effect of quizartinib on ABCG2-mediated MDR in cells overexpressing wild-type and mutant ABCG2 (R482T and R482G) *in vitro*. The effect of quizartinib on the efficacy of topotecan was analyzed using *in vivo* tumor xenograft models.

## RESULTS

### Quizartinib significantly potentiates the cytotoxicity of the wild type and 482-T mutant ABCG2 substrate anticancer drugs

**Figure 1:**
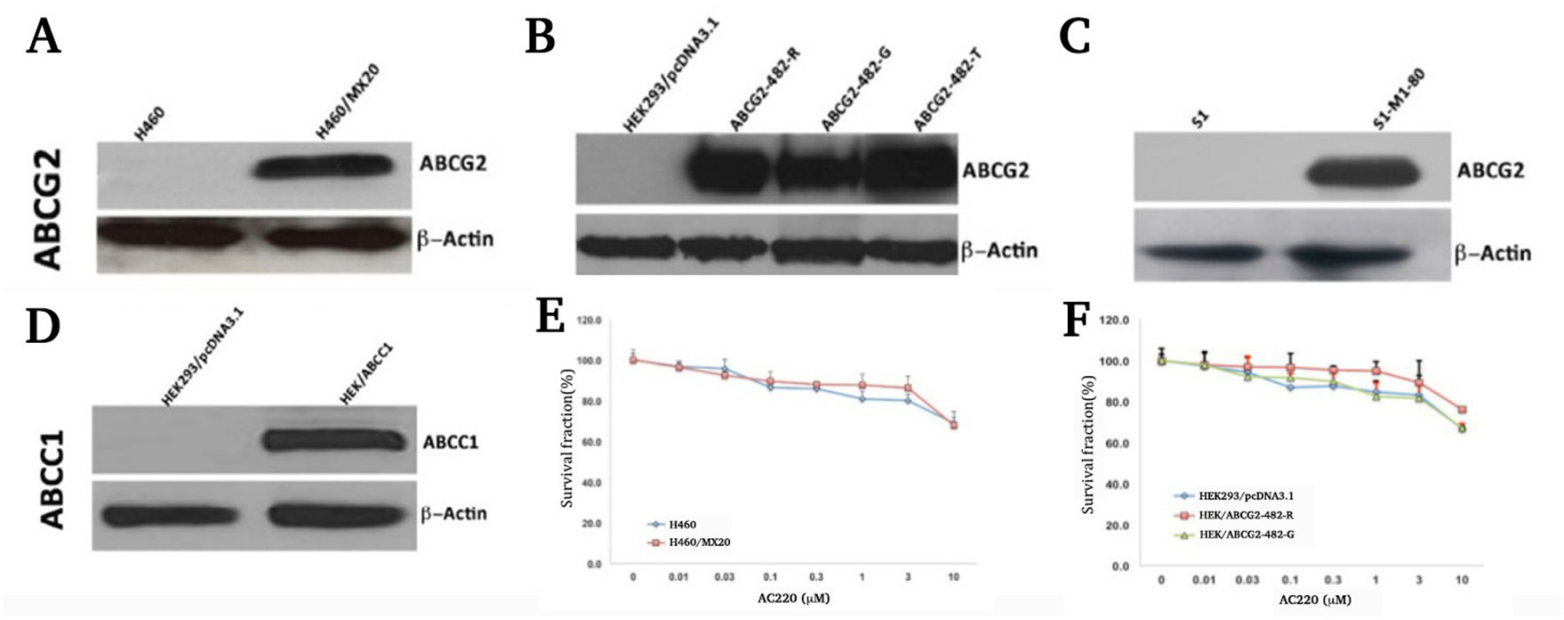
Western blotting shows expression of ABCG2 in H460, H460/MX20 cells **(A)**, HEK293/pcDNA3. 1, ABCG2-482-R, ABCG2-482-G and ABCG2-482-T cells **(B)**, S1 and S1-M1-80 cells **(C)**, and expression of ABCC1 in HEK/ABCC1 cells **(D)**. MTT cytotoxicity assay shows cell survival in H460 and H460/MX20 cells **(E)**, and HEK293/pcDNA3.1, ABCG2-482-R and ABCG2-482-G cells **(F)**. Data points represent the means ± SD of triplicate experiments.

The expression levels of ABCG2 (Figure 1A, 1B and 1C) or ABCC1 (Figure 1D) of the cell lines used in the study were confirmed by Western blotting before the MTT assay. In screening for ABC transporter inhibitors, we found that quizartinib can effectively reverse ABCG2-mediated MDR (Table 1). The cytotoxicity of quizartinib alone on ABCG2-overexpressing cell lines and ABCC1- overexpressing cell line were analyzed. At 3 μM, quizartinib has none to minimal toxicity to all the cell lines tested, with IC_50_ values of more than 10 μM (Figure 1E and 1F). Based on these results, the non-toxic concentrations of 0.75 and 3 μM were used in the following experiments.

**Table 1:** Quizartinib effectively sensitizes wild-type and mutant ABCG2-transfected cells to the substrate anticancer drugs

	IC_50_±SD (nM)			
Treatment	HEK293/pcDNA3.1	ABCG2-482R (WT)	ABCG2-482-G	ABCG2-482-T
Mitoxantrone	40.11±6.03 (1.00)^a^	466.33±40.17(11.63)^a^	1099.12±99.87(27.40)^a^	885.67±74.22(22.08)^a^
+AC220 (0.75 μM)	40.03±4.71 (1.00)	129.31±12.69 (3.22)^*^	888.76±99.93(22.16)	219.28±23.19(5.47)^*^
+AC220 (3 μM)	39.56±3.17 (0.99)	52.97±4.35 (1.32)^*^	669.82±49.34(16.70)^+^	63.98±9.19 (1.59)^*^
+FTC (3 μM)	37.57±4.29 (0.94)	46.67±4.23(1.16)^*^	65.89±6.43 (1.64)^*^	53.67±4.92 (1.34)^*^
+Novo (50 μM)	36.24±4.76 (0.90)	47.68±5.37 (1.19)^*^	727.54±49.32(18.13)^+^	676.55±66.34 (16.87)^+^
				
SN38	7.58±0.59 (1.00)^a^	209.29±26.24(27.61)^a^	272.54±26.32(35.96)^a^	209.07±17.45(27.58)^a^
+AC220 (0.75 μM)	7.33±0.76 (0.97)	70.25±5.67 (9.27)^*^	203.45±18.88(26.84)^+^	66.21±7.13(8.74)^*^
+AC220 (3 μM)	7.09±0.37 (0.94)	9.18±0.85 (1.21)^*^	171.23±19.21(22.59)^+^	10.12±1.89(1.34)^*^
+FTC (3 μM)	7.08±0.98 (0.93)	8.99±0.65 (1.19)^*^	8.98±0.87(1.18)^*^	8.87±0.79 (1.17)^*^
+Novo (50 μM)	7.78±0.70 (1.03)	8.67±0.83 (1.14)^*^	225.43±22.38 (29.74)^+^	188.32±17.86 (24.84)^+^
				
Topotecan	24.56 ±2.34 (1.0)^a^	334.76±33.45(13.63)^a^	367.98±31.34(14.98)^a^	311.43±37.32(12.68)^a^
+AC220 (0.75 μM)	23.78 ±2.54 (0.97)	204.26±20.09(8.32)^+^	336.89±30.37(13.72)	187.23±21.34 (7.62)^+^
+AC220 (3 μM)	22.88 ±2.65 (0.93)	38.97±3.96(1.59)^*^	342.01±31.78(13.93)	33.42±3.65(1.36)^*^
+FTC (3 μM)	20.99 ±1.76 (0.85)	27.03±2.65(1.10)^*^	30.41±2.78(1.24)^*^	28.09±2.65(1.14)^*^
+Novo (50 μM)	23.13±02.52 (0.94)	28.54±3.76 (1.16)^*^	332.57±35.54(13.54)	213.53±22.67(8.69)^+^
				
Cisplatin	2723.45±282.65 (1.00)^a^	2701.13±230.13(0.99)^a^	2667.53±220.32(0.98)^a^	2801.23±156.98 (1.03)^a^
+AC220 (0.75 μM)	2766.23±178.43(1.06)	2632.78±224.54(0.97)	2565.56±165.67(0.94)	2436.23±205.77 (0.89)
+AC220 (3 μM)	2664.65±177.87 (0.98)	2489.04±188.34(0.91)	2673.33±178.67 (0.98)	2458.76±176.45 (0.90)
+FTC (3 μM)	2599.97±210.32 (0.95)	2516.32±213.24(0.92)	2674.38±187.37 (0.98)	2558.69±212.34 (0.94)

Data represents the mean IC_50_ values for each cell line ± SD obtained from three independent sets of experiments. ^a^, values represent resistance fold (RF) that was determined by dividing the IC_50_ value of anticancer drug for HEK293/pcDNA3.1, ABCG2-482-R, ABCG2-482-G, ABCG2-482-T in the absence or presence of reversal agents by the IC_50_ value of respective anticancer drug for HEK293/pcDNA3.1 in the absence reversal agent. Cell survival assay was determined by the MTT assay as described in materials and methods. FTC was used as a positive control specific ABCG2 inhibitor. Novobiocin (Novo) was used as a positive control ABCG2 inhibitor to show inhibitory effects against wild type ABCG2 but weak on mutant ABCG2-482-T and ABCG2-482-G. ^*^, *P <* 0.01 and ^+^, *P* < 0.05 versus the control group.

HEK293 cells transfected with wild-type (ABCG2-482-R), mutant (ABCG2-482-G and ABCG2-482-T) ABCG2 showed significant resistance to mitoxantrone, SN-38 (active metabolite of topotecan) and topotecan compared to HEK293/pcDNA3.1 cells (Table 1). Quizartinib significantly increased the cytotoxicity of mitoxantrone, SN-38 and topotecan in wild-type and 482-T mutant ABCG2-transfected cells in a concentration dependent manner (Table 1). However, quizartinib showed only a moderate reversal effect in 482-G mutant ABCG2-transfected cells (Table 1). Additionally, the reversal effect of quizartinib at 3 μM on wild-type and 482T mutant ABCG2-mediated MDR was comparable to the effect produced by 3 μM of FTC, a known specific ABCG2 inhibitor (Table 1). Furthermore, the reversal effect of quizartinib on 482-T mutant ABCG2-mediated MDR is much better than that of novobiocin, an inhibitor of ABCG2 which more potently inhibits wild-type ABCG2 than 482-T and 482-R mutant ABCG2 (Table 1). However, quizartinib did not sensitize ABCG2-transfected cells to cisplatin, a non-substrate of ABCG2 (Table 1). The reversal effect of quizartinib was also analyzed in parental H460 and S1, drug selected wild-type ABCG2-overexpressing H460/MX20 and 482-G mutant ABCG2-overexpressing S1-M1-80 cells. We observed similar results that quizartinib significantly increased the cytotoxicity of mitoxantrone, SN-38 and topotecan in wild type ABCG2-overexpressing H460/MX20 (Table 2) and quizartinib only moderately sensitized 482-G mutant ABCG2-overexpressing S1-M1-80 cells (Table 2). However, quizartinib did not sensitize the parental HEK293/pcDNA3.1, H460 and S1 cells to ABCG2 substrate anticancer drugs (Table 2). In addition, we investigated the effect of quizartinib on ABCC1-overexpressing cells. Quizartinib was not able to reverse ABCC1-mediated MDR (Table 3).

**Table 2:** Quizartinib sensitizes ABCG2-overexpressing drug selected cell lines, to the ABCG2 substrate anticancer drugs

	IC_50_±SD (nM)	
Treatment	H460	H460/MX20 (WT-ABCG2)
Mitoxantrone	2.51±0.32 (1.00)^a^	1203.23±100.87 (479.37)^a^
+AC220 (0.75μM)	2.43±0.33 (0.97)	278.85±29.32 (111.10)^+^
+AC220 (3μM)	2.54±0.32 (1.01)	17.85±1.98 (7.11)^*^
+FTC (3μM)	2.23±0.35 (0.89)	22.63±2.78 (9.02)^*^
		
SN38	1.89±0.24 (1.00)^a^	918.23±63.67 (485.84)^a^
+AC220 (0.75μM)	1.87±0.23 (0.99)	302.12±40.89 (159.85)^+^
+AC220 (3μM)	1.83±0.21 (0.97)	17.72±2.50 (9.38)^*^
+FTC (3μM)	1.81±0.19 (0.96)	18.04±2.23 (9.54)^*^
		
Topotecan	8.43 ± 0.81 (1.0)^a^	1378.70 ± 135.24 (163.55)^a^
+AC220 (0.75μM)	8.40 ± 0.92 (1.0)	728.42 ± 63.23 (86.41)^+^
+AC220 (3μM)	8.17 ± 1.03 (0.97)	19.53 ± 2.27 (2.32)^*^
+FTC (3μM)	7.89 ± 1.12 (0.94)	16.56 ± 2.08 (1.96)^*^
		
Cisplatin	1822.50±143.38 (1.00)^a^	1873.27±179.34 (1.03)^a^
+AC220 (0.75μM)	1827.32±175.23 (1.00)	1798.45±147.96 (0.99)
+AC220 (3μM)	1803.65±166.56 (0.99)	1789.68±164.23 (0.98)
+FTC (3μM)	1799.58±185.44 (0.99)	1785.33±174.03 (0.98)

	IC_50_±SD (μM)	
Treatment	S1	S1-M1-80 (482-G)
Mitoxantrone	0.075±0.009 (1.00)^a^	27.12±3.12 (361.60)^a^
+AC220 (0.75 μM)	0.074±0.009 (0.99)	25.03±2.16 (333.73)
+AC220 (3 μM)	0.073±0.008 (0.97)	11.48±1.22 (153.07)^+^
+FTC (3 μM)	0.061±0.008 (0.81)	0.14±0.016 (1.87)^*^
		
SN38	1.03±0.093 (1.00)^a^	13.12±0.95 (12.74)^a^
+AC220 (0.75 μM)	0.98±0.079 (0.95)	11.15±1.37 (10.83)
+AC220 (3 μM)	0.93±0.085 (0.90)	8.28±0.94 (8.04)^+^
+FTC (3 μM)	0.88±0.063 (0.85)	1.93±0.18 (1.87)^*^
		
Cisplatin	1.96±0.15 (1.00)^a^	2.04±0.28 (1.04)^a^
+AC220 (0.75 μM)	1.88±0.19 (0.96)	1.98±0.21 (1.01)
+AC220 (3 μM)	1.90±0.17 (0.97)	1.95±0.17 (0.99)
+FTC (3 μM)	1.93±0.18 (0.98)	1.89±0.18 (0.96)

Data represents the mean IC_50_ values for each cell line ± SD obtained from three independent sets of experiments. ^a^, values represent resistance fold (RF) that was determined by dividing the IC_50_ value of anticancer drug for H460/ S1 and H460/MX20/ S1-M1-80 cells in the absence or presence of reversal agents by the IC_50_ value of respective anticancer drug for H460 cells in the absence reversal agent. Cell survival assay was determined by the MTT assay as described in materials and methods. FTC was used as a positive control specific ABCG2 inhibitor. ^*^, *P <* 0.01 and ^+^, *P* < 0.05 versus the control group.

**Table 3:** Quizartinib does not affect ABCC1-mediated MDR

	IC_50_ ± SD (nM)	
Treatments	HEK293/pcDNA3.1	HEK/ABCC1
Vincristine	15.21 ± 4.13 (1.0)	98.41 ± 7.22 (6.5)^a^
+ AC220 (3 μM)	16.33 ± 2.74 (1.1)	89.14 ± 10.43 (5.9)
+ ONO-1078 (3 μM)	11.80 ± 2.13 (0.8)	18.36 ± 4.43 (1.2)^*^

Data represents the mean IC_50_ (nM) values ± SD for each cell line obtained from three independent sets of experiments. ^a^, values represent resistance fold (RF) that was determined by dividing the IC_50_ value of anticancer drug for HEK293/pcDNA3.1 and HEK/ABCC1 in the absence or presence of reversal agents by the IC_50_ value of respective anticancer drug for HEK293/pcDNA3.1 in the absence reversal agent. Cell survival assay was determined by the MTT assay as described in materials and methods. ONO-1078 was used as a positive control inhibitor of ABCC1. ^*^, *P <* 0.01 versus the control group.

### Quizartinib enhances the intracellular accumulation of [^3^H]-mitoxantrone in cells overexpressing wild type and 482-T mutant ABCG2

**Figure 2:**
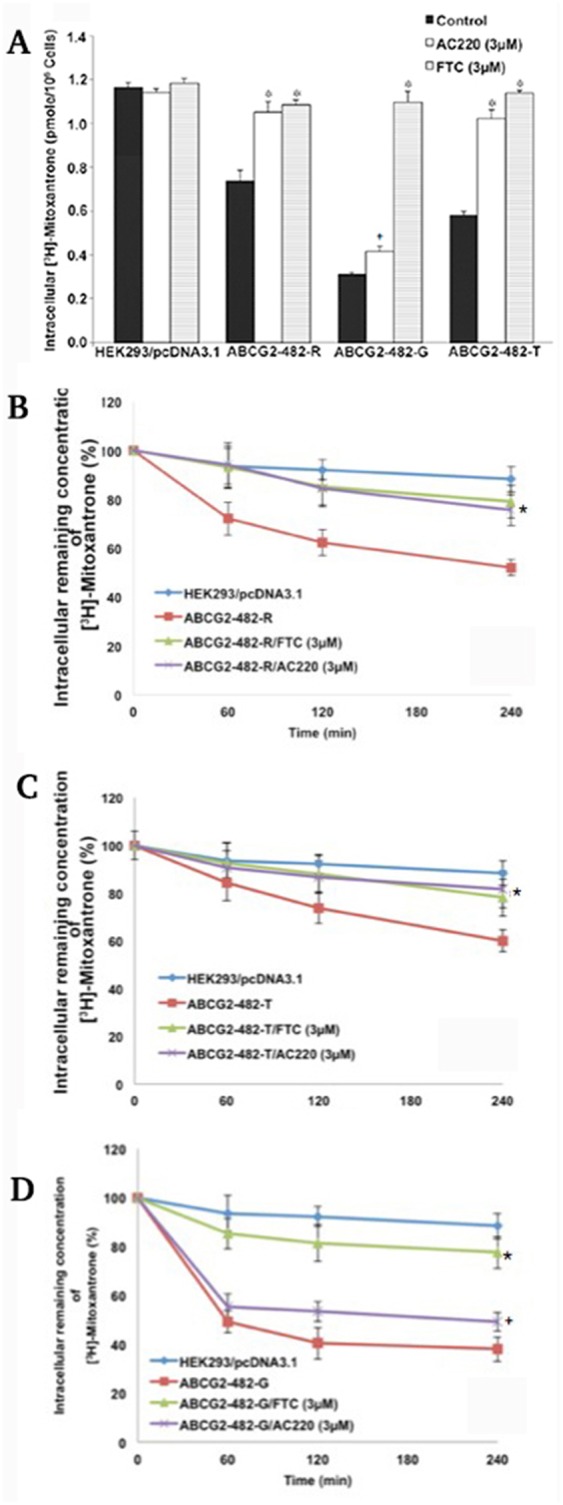
The effect of quizartinib on intracellular levels of [^3^H]-mitoxantrone **(A)** The accumulation of [^3^H]-mitoxantrone in HEK293/pcDNA3.1, ABCG2-482-R, ABCG2-482-G and ABCG2-482-T with quizartinib (AC220) or FTC treatment. Columns are the mean of triplicate determinations. ^*^, *P <* 0.05 and ^+^, 0.1 < *P* < 0.05 versus the control group. Experiments were performed three independent times, and a representative experiment is shown. **(B-D)** The effect of quizartinib on efflux of [^3^H]-mitoxantrone. The effect of quizartinib (3 μM) on retention of [^3^H]-mitoxantrone in HEK293/pcDNA3.1, ABCG2-482-R (B), ABCG2-482-T (C) and ABCG2-482-G (D). Data points represent the means ± SD. The figures are a representative of three independent experiments each done in triplicates. ^*^, *P <* 0.05 and ^**+**^, 0.1 < *P* < 0.05 versus the respective time point of control group.

To understand the mechanism of reversal, we examined the effect of quizartinib on the intracellular accumulation of ABCG2 substrate anticancer drug [^3^H]-mitoxantrone in ABCG2-overexpressing cells. The intracellular levels of [^3^H]-mitoxantrone were measured in cells with or without quizartinib. Quizartinib at 3 μM significantly increased the intracellular [^3^H]-mitoxantrone accumulation in both wild-type and 482-T mutant ABCG2-transfected cells (Figure 2), but only moderately increased the intracellular concentration of [^3^H]-mitoxantrone in the cells overexpressing 482-G mutant ABCG2. Neither quizartinib nor FTC significantly influenced the intracellular accumulation of [^3^H]-mitoxantrone in HEK293/pcDNA3.1 cells (Figure 2A). These results suggest that increased intracellular levels of [^3^H]-mitoxantrone in ABCG2-overexpressing cells may be a major mechanism of the reversal effect of quizartinib.

### Quizartinib decreases the efflux of [^3^H]-mitoxantrone in cells overexpressing ABCG2

Increased intracellular accumulation of [^3^H]-mitoxantrone by quizartinib may have two mechanisms. One possibility is that quizartinib may increase mitoxantrone uptake. Another one is that quizartinib may inhibit the mitoxantrone efflux. We performed an efflux assay and found that the extrusion rate of [^3^H]-mitoxantrone was significantly higher in ABCG2-482-R and ABCG2-482-T cells than in HEK293-pcDNA3.1 cells. Quizartinib at 3 μM time dependently blocked the efflux function of [^3^H]-mitoxantrone in wild-type (Figure 2B) and ABCG2-482-T cells (Figure 2C). However, quizartinib at 3 μM only moderately inhibited the efflux of [^3^H]-mitoxantrone in 482G mutant ABCG2-overexpressing cells (Figure 2D). Consistent with the cytotoxicity analysis, these data suggest that quizartinib increased the intracellular concentration of [^3^H]-mitoxantrone by inhibiting the efflux function of wild-type and 482-T mutant ABCG2.

### Quizartinib has no effect on the expression of either wild-type or 482 mutant ABCG2

**Figure 3:**
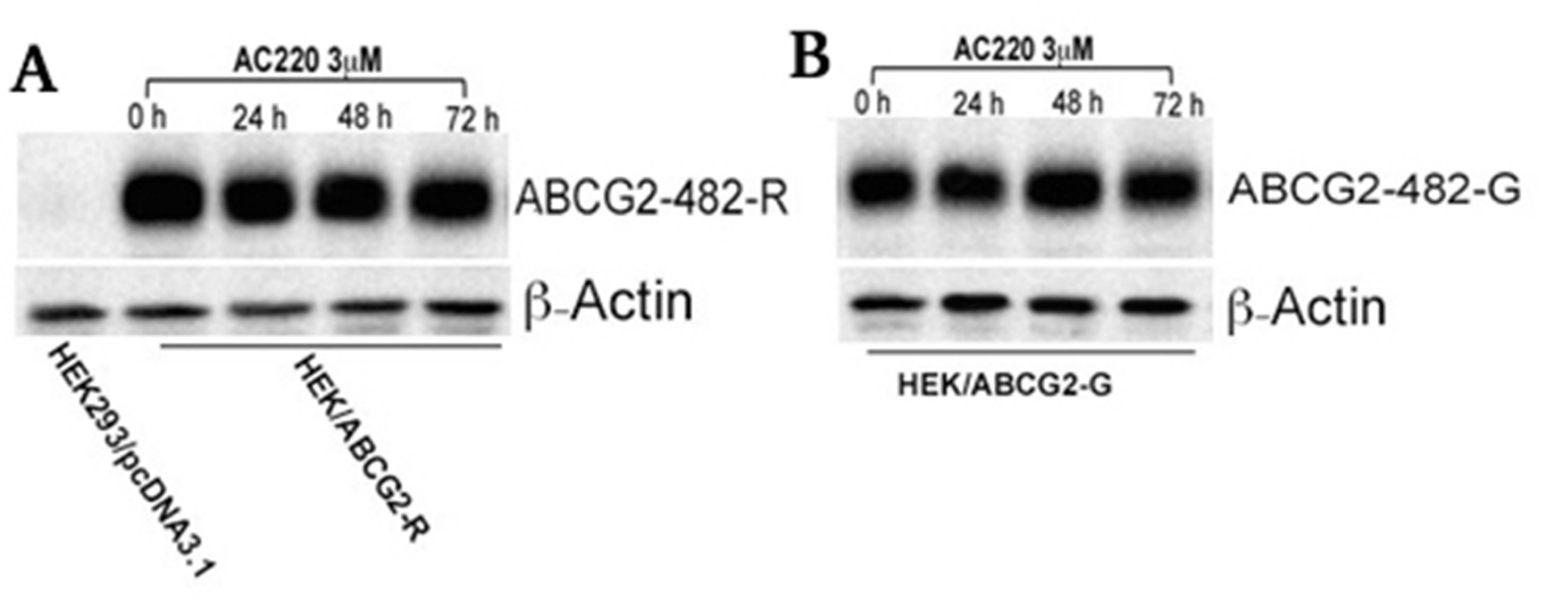
The effect of quizartinib on the expression of wild-type and 482-G mutant ABCG2 HEK293/pcDNA3.1, ABCG2-482-R **(A)**, and ABCG2-482-G **(B)** cells were treated with quizartinib (AC220) at 3 μM for 72 h. Western blotting was performed as described in “Materials and Methods”.

Reversal of ABCG2-mediated MDR by quizartinib could occur either by inhibiting the transporter function of ABCG2 or downregulation of the ABCG2 protein expression. To analyze the effect of quizartinib on the ABCG2 expression, we incubated ABCG2-overexpressing cells with quizartinib at 3 μM (24, 48 and 72 h). We found no change in the expression of wild-type (Figure 3A) and two mutant variants 482-G (Figure 3B) and 482-T (data not shown) upon quizartinib treatment. This result indicated that quizartinib does not decrease the expression of ABCG2 but rather, inhibits its efflux function.

### Molecular docking of quizartinib to human homology modeled ABCG2

**Figure 4:**
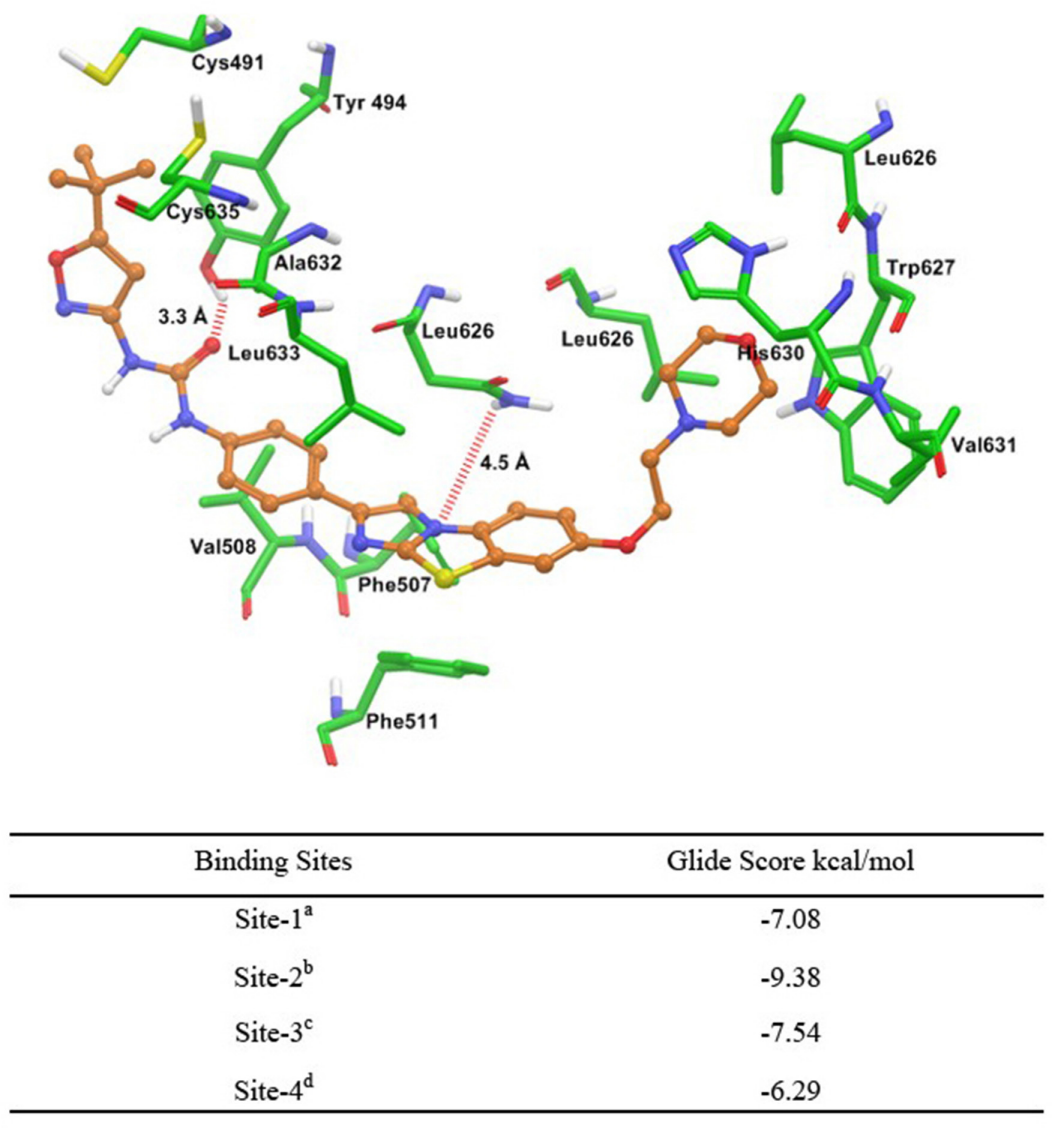
Glide predicted binding mode of quizartinib with homology modeled ABCG2 The docked conformation of quizartinib as ball and stick model is shown within the cavity of ABCG2. Important amino acids are depicted as sticks with the atoms colored as carbon – green, hydrogen – white, nitrogen – blue and oxygen – red, whereas quizartinib is shown with the same color scheme as above except carbon atoms are represented in orange and chlorine in dark green. Table shows the binding energies of quizartinib within each of the predicted sites of ABCG2. ^a^Site grid generated using Arg482; ^b^Site grid generated using Asn629; ^c^Site grid generated using Arg383; ^d^Site grid generated using Leu241 and Gly83.

To understand the plausible binding interaction of quizartinib to the homology model of human ABCG2 at molecular level, docking simulations were performed on all of the possible binding sites. The best docking score was found at site-2; therefore, the binding interaction model of quizartinib at site-2 is shown in Figure 4. These interactions suggested that quizartinib may bind to ABCG2 drug-binding site-2 with high affinity. The morpholinoethyl ring was stabilized through hydrophobic interactions with the side chains of residues Leu626, Trp627, His630, and Val631. The benzo[d]imidazo[2,1-b]thiazole group and the phenyl ring interacted with nearby uncharged residues Phe507, Val508, Phe511, Asn629, Ala632, and Leu633 through hydrophobic interactions. The N_3_ atom of the thiazole ring may form an electrostatic interaction with the side chain of Asn629 (N_3_···H_2_N-Asn629, 4.5 Å). The carbonyl oxygen atom of urea function may be involved in an electrostatic contact with the hydroxyl group of Tyr494 (CO···HO-Tyr494, 3.3 Å). The 5-*tert*-butylisoxazolyl group was stabilized by the side chains of Cys491, Cys635, Try494, and Ala632.

### Quizartinib potentiates the anticancer activity of topotecan in ABCG2-overexpressing tumor xenograft model

**Figure 5:**
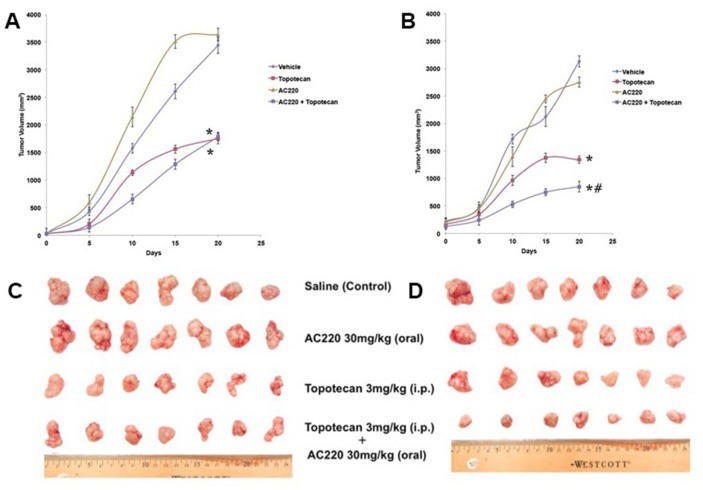
The effect of quizartinib on the tumor growth rate in H460 and H460/MX20 xenografts Changes in tumor volume with time in H460 xenograft **(A)** and H460/MX20 xenograft **(B)** are shown. Points represent mean tumor volume for each group after implantation. Each point on the line graph represents the mean tumor volume (mm^3^) at a particular day after implantation and the bars represent SD. Representative pictures of the excised H460 tumor **(C)** and H460/MX20 tumor **(D)** from different mice on the 20^th^ day after implantation are shown. The treatment regimens were as follows: Vehicle (q3d X 6), Topotecan (3 mg/kg, i.p., q3d X 6), Quizartinib (30 mg/kg, p.o., every 3^rd^ day) and Topotecan (3 mg/kg, i.p., q3d X 6) + Quizartinib (30 mg/kg, p.o., every 3^rd^ day, administered 1 h prior to Topotecan). ^*^, tumor volume was significantly decreased in comparison with vehicle group (p < 0.05). #, tumor volume was significantly decreased in comparison with topotecan alone group (p <0.05). Data are means ± SD for 7 animals. At least two independent experiments were carried out using athymic NCR nude mice.

**Figure 6:**
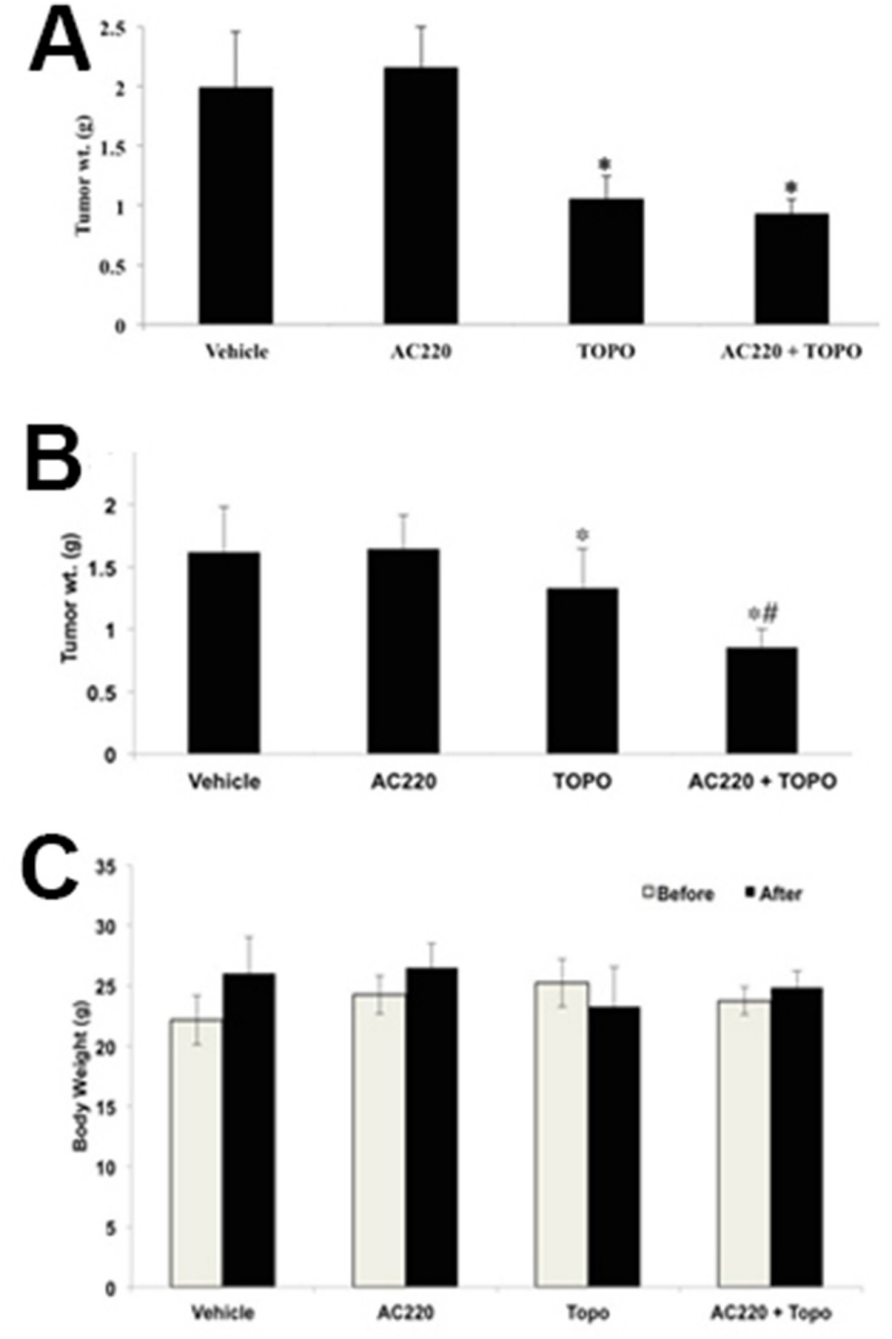
The effect of quizartinib on the tumor weight and body weight of H460 and H460/MX20 xenograft mice The bar graphs represent the mean tumor weight (n = 7) of the excised H460 tumor **(A)** and H460/MX20 tumor **(B)** from different mice. Changes of mean body weight before and after treatment are shown in the bar graph **(C)**. ^*^, tumor weight was significantly reduced in comparison with vehicle group (p < 0.05). #, tumor weight was significantly reduced in comparison with topotecan alone group (p <0.05).

The parental H460 and mitoxantrone selected ABCG2-overexpressing H460/MX20 xenograft MDR model in athymic nude mice was used to investigate the efficacy of quizartinib to reverse the resistance to topotecan *in vivo*. Quizartinib at 30 mg/kg oral dose was chosen based on our preliminary study (data not shown). This dose caused no visible toxicity or phenotypic changes in the male athymic NCR nude mice (data not shown). Topotecan at 3 mg/kg (i.p) showed appreciable tumor growth retardation in the parental H460 xenografts but not in H460/MX20 xenograft (Figure 5). The H460/MX20 tumor growth rate recorded in a period of 20 days was significantly slower in the quizartinib-topotecan combination group as compared to vehicle, quizartinib or topotecan alone groups (Figure 5B and 5D). In addition, quizartinib in combination with topotecan also produced a significant reduction in tumor weight at the end of the study in H460/MX20 xenograft (Figure 6B). It should be noted that quizartinib alone did not significantly decrease the growth rate of H460 and H460/MX20 xenografts (Figures 5 and 6). However, there was no significant difference between the effects of topotecan alone or combination of topotecan with quizartinib on H460 xenograft (Figure 5A and 6A). Topotecan with or without quizartinib did not cause significant weight loss (Figure 6C) or cause any mortality (data not shown).

## DISCUSSION

ABC transporters not only have a protective role against xenobiotics but also are involved in absorption and metabolism of certain chemotherapeutic agents [2, 3, 7, 8, 29-32]. Unfortunately, cancer cells utilize the protective function of ABC transporter as a survival mechanism. In the past three decades, extensive research has shown that overexpression of ABC transporters is related to MDR, and is one of the major factors resulting in chemotherapeutic failure.

The development of small molecule TKIs is a major therapeutic breakthrough in cancer treatment. Several small molecule TKIs have been approved for the treatment of various cancers, including imatinib (Gleevec, STI571), dasatinib (Sprycel, BMS-354825), nilotinib (Tasigna, AMN107) [34], erlotinib (Tarceva, OSI-774) [33], lapatinib (Tykerb, GW572016) [15]. These TKIs have shown clinical application in numerous cancers that are resistant to conventional treatment, such as different forms of leukemia, and lung cancer [33, 34].

Quizartinib is a potent and selective FLT3 kinase inhibitor [26]. Good aqueous solubility, better pharmacokinetic profile, and high efficacy as an antitumor agent in tumor xenograft studies have made quizartinib a promising clinical candidate. Recently, we showed that nilotinib reversed ABCB1- and ABCG2-mediated MDR to paclitaxel and DOX, respectively, in nude mouse tumor xenograft models [29]. We also showed that imatinib, erlotinib, lapatinib, and nilotinib reversed ABCC10-mediated MDR [22, 23]. In the current study, we examined the effect of quizartinib *in vitro* on wild type and 482-mutant ABCG2-mediated drug resistance in various cancer cells. We also analyzed the effect of quizartinib as an MDR reversal agent on ABCG2-overexpressing tumors *in vivo*.

In the cell viability assay, quizartinib potentiated the cytotoxicity of ABCG2 substrate anticancer drugs such as mitoxantrone, topotecan and SN-38 in wild-type ABCG2-transfected cells. Unlike previously characterized ABCG2 inhibitor novobiocin, quizartinib is able to reverse 482-T mutant ABCG2-mediated MDR. However, like novoviocin, quizartinib only moderately potentiated the cytotoxicity of substrate anticancer drugs in cells transfected with 482-G mutant ABCG2, which is different from FTC, another inhibitor of ABCG2 (Table 1). Similar results were obtained with drug selected cancer cells that acquired ABCG2-mediated resistance to mitoxantrone, such as H460/MX20 and S1-M1-80, overexpressing wild-type and 482-G mutant ABCG2, respectively. Moreover, quizartinib did not sensitize ABCC1-overexpressing HEK/ABCC1 cells. These results indicate that quizartinib specifically reverses the MDR associated with wild-type and 482-T mutant ABCG2. Furthermore, the moderately inhibitory effect of quizartinib to reverse the 482-G mutant ABCG2-mediated MDR suggests that the Arg-482 position is important for the inhibitory function of ABCG2 inhibitors.

To investigate the mechanism of the reversal of wild-type and 482-T mutant ABCG2-mediated MDR, we analyzed the effect of quizartinib on ABCG2-mediated drug transport. We performed drug accumulation assay with MDR cells with or without quizartinib. The results showed an increase in the accumulation of mitoxantrone in cells overexpressing wild-type and 482-T mutant ABCG2. Furthermore, it decreased the efflux rate of mitoxantrone in cells transfected with wild-type and 482-T mutant ABCG2 but only moderately inhibited the efflux of mitoxantrone in 482-G mutant ABCG2. The results of the accumulation and efflux experiments were consistent with the cytotoxic data, suggesting that quizartinib interacts synergistically with ABCG2 substrates and sensitizes ABCG2-overexpressing MDR cells to anticancer drugs. It is possible that the synergistic effect produced by quizartinib may be due to downregulation of ABCG2 expression following quizartinib treatment. To confirm this, ABCG2-overexpressing cells were treated with quizartinib, and the result suggested that there was no change in protein expression of ABCG2. Therefore, reversal effect of quizartinib on ABCG2 in MDR cells is not due to its effect on expression but most likely related to its inhibition of efflux function of ABCG2. Moreover, the moderate reversal activity of quizartinib for 482-G mutant ABCG2-mediated MDR in ABCG2-482-G and S1-M1-80 cells may be attributed to the position of the 482nd amino acid.

To identify the binding interactions of quizartinib with ABCG2, we performed a molecular docking study at highly scored druggable site on homology modeled, functionally active dimer form of human ABCG2. The inhibition of ABCG2 by the TKIs could be due to several reasons: (a) in general, TKIs are hydrophobic (calculated log P (ClogP) value ranges from 3 to 6) and the drug binding site of ABCB1 and ABCG2 is highly hydrophobic [35], and (b) both ATP binding sites of TKs and transmembrane domains of human ABCB1 and ABCG2 are hydrophobic in nature. The calculated logP value of quizartinib was found to be 5.5. Quizartinib binded to large hydrophobic pockets in ABCG2 and these binding profiles may be associated with its highly hydrophobic properties. Aromatic rings in quizartinib such as tricyclic benzo[d]imidazo[2,1-b]thiazole ring, phenyl ring and isoxazolyl ring may be essential features for binding to ABC transporters based on a previous QSAR study [35]. Though docking is a useful tool in understanding ligand-protein interactions, the present study involves ABCG2, which is particularly challenging since ABCG2 may be active only in dimer or oligomer form. Hence, until the co-crystal structure studies are performed on quizartinib-ABCG2 complex, the present docking conformation of quizartinib could serve as a guide for further development of this class of ABCG2 inhibitors.

Our H460/MX20 xenograft model showed significant resistance to topotecan, an ABCG2 substrate anticancer drug. Quizartinib significantly enhanced the anticancer activity of topotecan in H460/MX20 xenograft (Figures 5 and 6). The positive outcome implies that quizartinib can be combined with conventional ABCG2 substrate chemotherapeutic drugs as well as other TKIs that are substrates of ABCG2. Quizartinib alone did not show antitumor activity *in vivo* in both H460 and H460/MX20 xenograft models. Previous reports have shown that second generation bis-aryl urea FLT3 inhibitor, quizartinib, potently inhibits ABCG2 at clinically used concentrations and thus may sensitize AML cells overexpressing ABCG2 transporter to ABCG2 substrate anticancer drugs [25, 28]. These results indicate that quizartinib potentiates the anticancer effect of topotecan in H460/MX20 xenograft model at concentrations that are clinically achievable. To our knowledge, this may be the first report that quizartinib could reverse ABCG2-mediated MDR in an *in vivo* model.

In conclusion, quizartinib effectively inhibits wild-type and 482-T mutant ABCG2 efflux function, and reverses wild-type and 482-T mutant ABCG2-mediated MDR without affecting the expression of the transporter. Quizartinib potentiates the antitumor effect of topotecan in ABCG2-overexpressing H460/MX20 mouse xenografts. These results suggest that quizartinib could be used to augment conventional chemotherapeutic drugs as well as other TKIs that are substrates of ABCG2, in patients with MDR mediated by ABCG2 transporter.

## MATERIALS AND METHODS

### Chemicals

[^3^H]-Mitoxantrone (4 Ci/mmol) was purchased from Moravek Biochemicals, Inc (Brea, CA). Dulbecco’s modified Eagle’s medium (DMEM), fetal bovine serum (FBS), penicillin/streptomycin and trypsin 0.25% were purchased from Hyclone (Waltham, MA). The monoclonal antibodies BXP-21 (against ABCG2), sc-8432 (against actin) and the secondary horseradish peroxidase-labeled anti-mouse IgG were purchased from Santa Cruz Biotechnology, Inc. (Santa Cruz, CA). Anti β actin mAb (mouse) was purchased from Genescript (Piscataway, NJ). Fumitremorgin C (FTC) was synthesized by Thomas McCloud, Developmental Therapeutics Program, and Natural Products Extraction Laboratory, NIH (Bethesda, MD) and was a gift from Drs. Susan Bates and Robert Robey (NCI, NIH). ONO-1078 (specific ABCC1 inhibitor) was a gift from Dr. Shin-ichi Akiyama (Kagoshima, Japan). Quizartinib was purchased from Chemietek (Indianapolis, IN). Topotecan was purchased from LC laboratories (Woburn, MA). Mitoxantrone, SN-38, cisplatin, MTT (3-(4,5-dimethylthiazol-yl)-2,5-diphenyltetrazolium bromide), dimethyl sulfoxide (DMSO) and other chemicals were obtained from Sigma Chemical Co. (St. Louis, MO).

### Cell lines

HEK293/pcDNA3.1, ABCG2-482-R, ABCG2-482-G, ABCG2-482-T and HEK/ABCC1 cell lines were established by selection with G418 (2 mg/mL) after transfecting HEK293 cell line with either an empty pcDNA3.1 vector or pcDNA3.1 vector containing a full length *ABCG2* with Arg, Gly or Thr at position 482, respectively, or *ABCC1*, and were cultured in medium with 2 mg/mL of G418. The H460, S1, ABCG2-overexpressing H460/MX20 and S1-M1-80 cells were kindly provided by Drs. Susan Bates and Robert Robey (NCI, NIH, Bethesda). All cells were grown as adherent monolayer in drug-free culture media for at least 2 weeks before assay. All cell lines were cultured at 37°C with 5% CO_2_ and DMEM containing 10% FBS and 1% penicillin/streptomycin.

### Cytotoxicity evaluation by MTT assay

Briefly, cells were harvested and re-suspended at a final concentration of 3×10^3^ cells/well for H460 and S1 cells, and 5×10^3^ cells/well for H460/MX20, ABCG2-482-R, S1-M1-80, HEK293/pcDNA3.1, ABCG2-482-G, ABCG2-482-T, and HEK/ABCC1 cells. Cells were seeded evenly into (160 μL/well) 96-well plates. After incubating for 24 h at 37°C, 20 μL of various concentrations of the appropriate anticancer drug were added (20 μL of fixed concentration of the reversal compounds were added 1 h prior to the addition of anticancer drug). Subsequently the cells were incubated at 37°C for 72 h. After 72 h, 20 μL MTT (4 mg/ml) was added to each well. The plates were incubated at 37°C for 4 h. The MTT/medium was removed from each well without disturbing the cells, and 100 μL of DMSO was added. Finally, the absorbance was read at 570 nm with the help of Glomax Multi+ detection system (Promega, Madison, WI).

### [^3^H]-Mitoxantrone accumulation and efflux assay

The parental HEK293/pcDNA3.1, ABCG2-482-R, ABCG2-482-G, and ABCG2-482-T cells were trypsinized and two aliquots (12 × 10^6^ cells) from each cell line were suspended in the medium, pre-incubated with or without reversal agent at 37°C for 1 h. Subsequently, cells were suspended in the medium containing 0.1 μM [^3^H]-mitoxantrone with or without the reversal agent at 37°C for 2 h. The cells were washed with ice cold phosphate-buffered saline (PBS) three times and radioactivity was measured. Efflux assay was adopted from our previous study [23]. Briefly, parental HEK293-pcDNA3.1, and ABCG2-482-R, ABCG2-482-G, and ABCG2-482-T cells were trypsinized and two aliquots (48 × 10^6^ cells) from each cell line were suspended in the medium, pre-incubated with or without reversal agent at 37°C for 1 h. Subsequently, cells were suspended in the medium containing 0.1 μM [^3^H]-mitoxantrone with or without reversal agent at 37°C for 2 h. The cells were washed with ice cold PBS three times, and then suspended in fresh medium with or without quizartinib or FTC at 37°C. Aliquots (1 × 10^6^ cells) were collected at various time points (0, 60, 120, and 240 min). Radioactivity was measured in a Packard TRI-CARB^®^ 1900CA liquid scintillation analyzer from Packard Instrument Company, Inc (Downers Grove, IL).

### Western blot analysis

Cell lysates were prepared as described previously [36]. Equal amounts of total cell lysates (30 μg protein) were resolved by sodium dodecyl sulfate polyacrylamide gel electrophoresis (SDS-PAGE) and electrophoretically transferred onto polyvinylidene fluoride (PVDF) membranes. After incubation in a blocking solution in TBST buffer (10 mM Tris–HCl, pH 8.0, 150 mM NaCl, and 1% Tween 20) for 1 h at room temperature, the membranes were immunoblotted overnight with primary monoclonal antibodies against β actin at 1:1000 dilution or ABCG2 at 1:200 dilution at 4°C, and were then further incubated for 2 h at room temperature with horseradish peroxide (HRP)-conjugated secondary antibody (1:1000 dilution). The protein–antibody complex was detected by enhanced chemiluminescence detection system (Amersham, NJ).

### Molecular modeling for ABCG2

#### Ligand structure preparation

Quizartinib structure was built using the fragment dictionary of Maestro v9.0 and energy minimized by Macromodel program v 9.7 (Schrödinger, Inc., New York, NY, 2009) using the OPLSAA force field with the steepest descent followed by truncated Newton conjugate gradient protocol. The low-energy 3D structures of quizartinib were generated by LigPrep v2.3 and the parameters were defined based on different protonation states at physiological pH±2, all possible tautomers and ring conformations. Ligand structures obtained from the LigPrep v2.3 run were further used for generating 100 ligand conformations for each protonated structure using the default parameters of mixed torsional/low-mode sampling function. The conformations were filtered with a maximum relative energy difference of 5 kcal/mol to exclude redundant conformers. The output conformational search (C search) file containing at most 100 unique conformers of quizartinib were used as input for docking simulations into binding site of homology modeled human ABCG2.

#### Protein structure preparation and docking protocol

Homology model of ABCG2 was built based on the mouse p-glycoprotein (PDB ID: 3G5U) [37] as template and has been generated and provided as the PDB file to us by Rosenberg et al [38, 39]. The homology model of ABCG2 PDB file was energy minimized before initiating grid preparation. To identify the druggable sites on ABCG2 homology model, we have generated various grids based on the following residues as centroids, for example, Arg482 (grid 1), Asn629 (grid 2), Arg383 (grid 3) and Leu241 along with Gly83 (grid 4). The choices of these residues were based on their involvement in ABCG2 function as determined through mutational experiments [40, 41]. All docking calculations were performed using the “Extra Precision” (XP) mode of Glide docking program v6.0 (Schrödinger, LLC, New York, NY, 2013) and the default parameters. The top-scoring pose-ABCG2 complex structures were then used for graphical analysis. All computations were carried out on a Dell Precision 490n dual processor with Linux OS (Ubuntu 12.04 LTS).

### Animals

Male athymic NCR (nu/nu) nude mice (13 – 15 g, age 4 – 5 wk), were purchased from the Taconic Farms (NCRNU-M, Homozygous, Albany, NY) and were used for tumor xenograft. All the animals were maintained on an alternating 12 h light/dark cycle with free access to water and rodent chow ad libitum. The mice were maintained at the St. John’s University Animal Facility and were monitored closely for tumor growth by palpation and visual examination. Institutional Animal Care & Use Committee (IACUC) of St. John’s University approved this project, and the research was conducted in compliance with the Animal Welfare Act and other federal statutes.

### Nude mice ABCG2-overexpressing tumor xenograft model

Briefly, H460 (1 x 10^6^) and H460/MX20 (3 x 10^6^) cells were injected s.c. under the armpits. Tumors that fail to reach a volume of 30 mm^3^ at the start of treatment were not used in the study. The mice were randomized into four groups (n=7) and treated with one of the following regimens: (a) vehicle (10% N-methyl-pyrrolidinone, 90% polyethylene glycol 300) (q3d X 6), (b) Topotecan (3 mg/kg, i.p., q3d X 6), (c) quizartinib dissolved in 10% N-methyl-pyrrolidinone, 90% polyethylene glycol 300 (30 mg/kg, p.o., every 3^rd^ day), and (d) Topotecan (3 mg/kg, i.p., q3d X 6) + quizartinib (30 mg/kg, p.o., every 3^rd^ day, given 1 h before giving Topotecan). Topotecan for injection was prepared by dissolving it in sterile water. Tumor volume was measured using calipers and body weights were recorded. The two perpendicular diameters of tumors (termed A and B) were recorded every 3 days and tumor volume (V) was estimated according to the formula published previously. At the end of the study, animals were euthanized by carbon dioxide, tumor tissue were excised and weighed.

$$V=\frac{\pi }{6}{\left(\frac{A+B}{2}\right)}^{3}$$

### Statistical analysis

Differences of the parameters between two groups were analyzed by two-tailed Student’s *t* test. *P* < 0.05 was considered as statistically significant.

## References

[1] Szakács G, Paterson JK, Ludwig JA, Booth-Genthe C, Gottesman MM (2006). Targeting multidrug resistance in cancer. Nature Reviews Drug Discovery.

[2] Gottesman MM, Fojo T, Bates SE (2002). Multidrug Resistance in Cancer: Role of Atp-Dependent Transporters. Nature Reviews Cancer.

[3] Deeley RG, Westlake C, Cole SP (2006). Transmembrane transport of endo- and xenobiotics by mammalian ATP-binding cassette multidrug resistance proteins. Physiol Rev.

[4] Akiyama S, Chen ZS, Kitazono M, Sumizawa T, Furukawa T, Aikou T (1999). Mechanisms for resistance to anticancer agents and the reversal of the resistance. [Article in Japanese]. Hum Cell.

[5] Mao Q, Unadkat JD (2005). Role of the breast cancer resistance protein (ABCG2) in drug transport. AAPS J.

[6] Ambudkar SV, Dey S, Hrycyna CA, Ramachandra M, Pastan I, Gottesman MM (1999). Biochemical, cellular, and pharmacological aspects of the multidrug transporter. Annu Rev Pharmacol Toxicol.

[7] Borst P, Elferink RO (2002). Mammalianabc Transporters Inhealth Anddisease. Annual Review of Biochemistry.

[8] Dean M (2001). The Human ATP-Binding Cassette (ABC) Transporter Superfamily. Genome Research.

[9] Tiwari AK, Sodani K, Dai CL, Ashby CR, Chen ZS (2011). Revisiting the ABCs of multidrug resistance in cancer chemotherapy. Curr Pharm Biotechnol.

[10] Kumar P, Zhang DM, Degenhardt K, Chen ZS (2012). Autophagy and transporter-based multi-drug resistance. Cells.

[11] Dean M, Annilo T (2005). Evolution of the Atp-Binding Cassette (Abc) Transporter Superfamily in Vertebrates. Annual Review of Genomics and Human Genetics.

[12] Gottesman MM, Ambudkar SV (2001). Overview: ABC transporters and human disease. J Bioenerg Biomembr.

[13] Sodani K, Patel A, Kathawala RJ, Chen ZS (2012). Multidrug resistance associated proteins in multidrug resistance. Chin J Cancer.

[14] Szakacs G, Varadi A, Ozvegy-Laczka C, Sarkadi B (2008). The role of ABC transporters in drug absorption, distribution, metabolism, excretion and toxicity (ADME-Tox). Drug Discov Today.

[15] Shi Z, Peng XX, Kim IW, Shukla S, Si QS, Robey RW, Bates SE, Shen T, Ashby CR, Fu LW, Ambudkar SV, Chen ZS (2007). Erlotinib (Tarceva, OSI-774) antagonizes ATP-binding cassette subfamily B member 1 and ATP-binding cassette subfamily G member 2-mediated drug resistance. Cancer Res.

[16] Dai CL, Tiwari AK, Wu CP, Su XD, Wang SR, Liu DG, Ashby CR, Huang Y, Robey RW, Liang YJ, Chen LM, Shi CJ, Ambudkar SV (2008). Lapatinib (Tykerb, GW572016) reverses multidrug resistance in cancer cells by inhibiting the activity of ATP-binding cassette subfamily B member 1 and G member 2. Cancer Res.

[17] Hegedus C, Ozvegy-Laczka C, Szakacs G, Sarkadi B (2009). Interaction of ABC multidrug transporters with anticancer protein kinase inhibitors: substrates and/or inhibitors?. Curr Cancer Drug Targets.

[18] Erlichman C, Boerner SA, Hallgren CG, Spieker R, Wang XY, James CD, Scheffer GL, Maliepaard M, Ross DD, Bible KC, Kaufmann SH (2001). The HER tyrosine kinase inhibitor CI1033 enhances cytotoxicity of 7-ethyl-10-hydroxycamptothecin and topotecan by inhibiting breast cancer resistance protein-mediated drug efflux. Cancer research.

[19] Ozvegy-Laczka C, Cserepes J, Elkind NB, Sarkadi B (2005). Tyrosine kinase inhibitor resistance in cancer: role of ABC multidrug transporters. Drug resistance updates.

[20] Tiwari AK, Sodani K, Wang SR, Kuang YH, Ashby CR, Chen X, Chen ZS (2009). Nilotinib (AMN107, Tasigna) reverses multidrug resistance by inhibiting the activity of the ABCB1/Pgp and ABCG2/BCRP/MXR transporters. Biochem Pharmacol.

[21] Shukla S, Robey RW, Bates SE, Ambudkar SV (2009). Sunitinib (Sutent(R), SU11248), a small-molecule receptor tyrosine kinase inhibitor, blocks function of the ABC transporters, P-glycoprotein (ABCB1) and ABCG2. Drug Metab Dispos.

[22] Shen T, Kuang YH, Ashby CR, Lei Y, Chen A, Zhou Y, Chen X, Tiwari AK, Hopper-Borge E, Ouyang J, Chen ZS (2009). Imatinib and nilotinib reverse multidrug resistance in cancer cells by inhibiting the efflux activity of the MRP7 (ABCC10). PLoS One.

[23] Kuang YH, Shen T, Chen X, Sodani K, Hopper-Borge E, Tiwari AK, Lee JW, Fu LW, Chen ZS (2010). Lapatinib and erlotinib are potent reversal agents for MRP7 (ABCC10)-mediated multidrug resistance. Biochem Pharmacol.

[24] Sun YL, Kumar P, Sodani K, Patel A, Pan Y, Baer MR, Chen ZS, Jiang WQ (2014). Ponatinib enhances anticancer drug sensitivity in MRP7-overexpressing cells. Oncology reports.

[25] Zarrinkar PP, Gunawardane RN, Cramer MD, Gardner MF, Brigham D, Belli B, Karaman MW, Pratz KW, Pallares G, Chao Q, Sprankle KG, Patel HK, Levis M (2009). AC220 is a uniquely potent and selective inhibitor of FLT3 for the treatment of acute myeloid leukemia (AML). Blood.

[26] Chao Q, Sprankle KG, Grotzfeld RM, Lai AG, Carter TA, Velasco AM, Gunawardane RN, Cramer MD, Gardner MF, James J, Zarrinkar PP, Patel HK, Bhagwat SS (2009). Identification of N-(5-tert-butyl-isoxazol-3-yl)-N'-{4-[7-(2-morpholin-4-yl-ethoxy)imidazo[2,1-b][1, 3]benzothiazol-2-yl]phenyl}urea dihydrochloride (AC220), a uniquely potent, selective, and efficacious FMS-like tyrosine kinase-3 (FLT3) inhibitor. Journal of medicinal chemistry.

[27] Gunawardane RN, Nepomuceno RR, Rooks AM, Hunt JP, Ricono JM, Belli B, Armstrong RC (2013). Transient exposure to quizartinib mediates sustained inhibition of FLT3 signaling while specifically inducing apoptosis in FLT3-activated leukemia cells. Molecular cancer therapeutics.

[28] Bhullar J, Natarajan K, Shukla S, Mathias TJ, Sadowska M, Ambudkar SV, Baer MR (2013). The FLT3 inhibitor quizartinib inhibits ABCG2 at pharmacologically relevant concentrations, with implications for both chemosensitization and adverse drug interactions. PLoS One.

[29] Tiwari AK, Sodani K, Dai CL, Abuznait AH, Singh S, Xiao ZJ, Patel A, Talele TT, Fu L, Kaddoumi A, Gallo JM, Chen ZS (2013). Nilotinib potentiates anticancer drug sensitivity in murine ABCB1-, ABCG2-, and ABCC10-multidrug resistance xenograft models. Cancer Lett.

[30] Borst P, Evers R, Kool M, Wijnholds J (2000). A family of drug transporters: the multidrug resistance-associated proteins. J Natl Cancer Inst.

[31] Chen ZS, Guo Y, Belinsky MG, Kotva E, Kruh GD (2005). Transport of bile acids, sulfated steroids, estradiol 17-beta-D-glucuronide, and leukotriene C4 by human multidrug resistance protein 8 (ABCC11). Mol Pharmacol.

[32] Chen ZS, Hopper-Borge E, Belinsky MG, Shchaveleva I, Kotova E, Kruh GD (2003). Characterization of the transport properties of human multidrug resistance protein 7 (MRP7, ABCC10). Mol Pharmacol.

[33] Al Olayan A, Al Hussaini H, Jazieh AR (2012). The roles of epidermal growth factor receptor (EGFR) inhibitors in the management of lung cancer. J Infect Public Health.

[34] Bisen A, Claxton DF (2013). Tyrosine kinase targeted treatment of chronic myelogenous leukemia and other myeloproliferative neoplasms. Adv Exp Med Biol.

[35] Nicolle E, Boumendjel A, Macalou S, Genoux E, Ahmed-Belkacem A, Carrupt PA, Di Pietro A (2009). QSAR analysis and molecular modeling of ABCG2-specific inhibitors. Advanced drug delivery reviews.

[36] Sodani K, Tiwari AK, Singh S, Patel A, Xiao ZJ, Chen JJ, Sun YL, Talele TT, Chen ZS (2012). GW583340 and GW2974, human EGFR and HER-2 inhibitors, reverse ABCG2- and ABCB1-mediated drug resistance. Biochem Pharmacol.

[37] Aller SG, Yu J, Ward A, Weng Y, Chittaboina S, Zhuo R, Harrell PM, Trinh YT, Zhang Q, Urbatsch IL, Chang G (2009). Structure of P-glycoprotein reveals a molecular basis for poly-specific drug binding. Science.

[38] Rosenberg MF, Bikadi Z, Chan J, Liu X, Ni Z, Cai X, Ford RC, Mao Q (2010). The human breast cancer resistance protein (BCRP/ABCG2) shows conformational changes with mitoxantrone. Structure.

[39] Hazai E, Bikadi Z (2008). Homology modeling of breast cancer resistance protein (ABCG2). J Struct Biol.

[40] Robey RW, Honjo Y, Morisaki K, Nadjem TA, Runge S, Risbood M, Poruchynsky MS, Bates SE (2003). Mutations at amino-acid 482 in the ABCG2 gene affect substrate and antagonist specificity. British journal of cancer.

[41] Mitomo H, Kato R, Ito A, Kasamatsu S, Ikegami Y, Kii I, Kudo A, Kobatake E, Sumino Y, Ishikawa T (2003). A functional study on polymorphism of the ATP-binding cassette transporter ABCG2: critical role of arginine-482 in methotrexate transport. Biochem J.

